# Induction of systemic and therapeutic antitumor immunity using intratumoral injection of bone-marrow derived dendritic cells genetically modified to express interleukin 12 combined with anti-CTLA-4 antibody

**DOI:** 10.1186/2051-1426-3-S2-P243

**Published:** 2015-11-04

**Authors:** Marimo Sato-Matsushita, Yoshihiro Hayakawa, Asuka Asami, Setsuko Nakayama, Hideaki Tahara

**Affiliations:** 1 The University of Tokyo, Minato-city, Japan; 2 The University of Toyama, Toyama-city, Japan

## 

We have been involved in the development of cancer immunotherapy using dendritic cells (DCs) manipulated to induce better immune responses. Our strategies include the usage of the agent to induce desirable maturation of DCs in culture and the genetic modification of DCs to have better function in situ. We have been developing immune-gene therapy with DCs genetically modified to continuously express IL-12. In this study, we investigated whether bone marrow-derived dendritic cells adenovirally transduced with genes encoding murine IL-12 combined with anti-CTLA-4 monoclonal antibody (Ad-IL-12-DCs) have significant therapeutic benefits for anti-tumor immunotherapy. Ad-IL-12-DCs was confirmed to express bioactive IL-12 proteins at high levels, and treatment with Ad-IL-12-DCs and anti-CTLA-4 monoclonal antibody showed enhanced anti-tumor effects and induced tumor-specific cytotoxic T lymphocyte responses *in vivo*. As a consequence of these stimulatory effects, combined treatment with DC vaccine and anti-CTLA-4 monoclonal antibody accomplished increased anti-tumor effects when compared with either mono-therapy. The information related to these trials would be useful to develop effective immunotherapy against cancer.

